# Physicochemical and Microbiological Characterization of Cilentana Goat Cheese

**DOI:** 10.3390/foods15183226

**Published:** 2026-09-12

**Authors:** Loriana Casalino, Marika Di Paolo, Marica Egidio, Giulia Polizzi, Maria Francesca Peruzy, Roberta Mazzocca, Emanuele D’Anza, Raffaele Marrone

**Affiliations:** 1 Department of Economics, Statistics and Business, Universitas Mercatorum, Piazza Mattei, 10, 00186 Rome, RM, Italy; loriana.casalino@studenti.unimercatorum.it; 2 Department of Veterinary Medicine and Animal Production, University of Napoli Federico II, 80137 Naples, NA, Italy; marika.dipaolo@unina.it (M.D.P.); mariafrancesca.peruzy@unina.it (M.F.P.); robertamazzocca27@gmail.com (R.M.); emanuele.danza@unina.it (E.D.); raffaele.marrone@unina.it (R.M.); 3 Department of Veterinary Medical Sciences, Alma Mater Studiorum—University of Bologna, Via Tolara di Sopra, 50, Ozzano dell’Emilia, 40064 Bologna, BO, Italy; giulia.polizzi3@unibo.it

**Keywords:** fatty acid profile, ripening, raw milk, cheese microbiology, traditional cheese

## Abstract

Cilentana goat cheese is a traditional dairy product made from raw goat’s milk in the Cilento region (Southern Italy). This study investigated microbiological, physicochemical, and lipid changes occurring during cheese-making and 90 days of ripening. Four independent batches were produced from raw Cilentana goat milk and sampled as milk, curd, and cheeses at 0, 30, 60, and 90 days for microbiological, physicochemical, fatty acid, lipolysis, oxidative stability, and color analyses. Overall, ripening promoted the establishment of a dominant lactic acid bacteria population, with mesophilic lactococci and lactobacilli reaching approximately 8 and 7.5 Log CFU/g, respectively, while *E. coli* and coagulase-positive staphylococci progressively declined below detectable or regulatory levels by day 60. *Salmonella* spp. and *Listeria monocytogenes* were never detected, although coliforms persisted throughout ripening, highlighting the need for improved raw milk hygiene. From a chemical perspective, lipolysis progressed during ripening, with free fatty acids (FFAs) significantly increasing after 90 days, while malondialdehyde levels remained low and unchanged, indicating limited lipid oxidation. The fatty acid profile and nutritional quality indices remained stable throughout ripening. In conclusion, the data collected provided a better characterization of this traditional cheese and can be used for broadening knowledge of raw goat’s milk cheeses.

## 1. Introduction

In recent years, interest in goat farming has increased, leading to renewed recognition of the species’ economic and productive importance within the livestock sector [1]. Although for a period this species was largely neglected, leading to the endangerment of several local breeds, there has been a renewed interest in recent years, driven by the growing emphasis on biodiversity preservation, sustainability, and the valorization of traditional agricultural systems. In Italy, such systems are especially prevalent in the southern regions and islands, where goat farming is closely tied to traditional agro-pastoral landscapes and relies heavily on autochthonous breeds [2]. One such breed is the Cilentana goat, native to the Cilento region in southern Italy, an area bordered by the Sele River to the north, the Vallo di Diano to the east, and the Tyrrhenian Sea to the south and west. Beyond its importance for the conservation of local genetic resources, this autochthonous breed supports a traditional dairy production system based on cheeses with distinctive local quality traits. The technological suitability of its milk for cheesemaking is closely related to its physicochemical composition, consisting of approximately 87% water, 4% fat, 4% lactose, 3.5% protein and 1% ash [3]. Its lipid fraction, composed predominantly of triacylglycerols (approximately 98%) together with minor constituents including phospholipids, cholesterol and free fatty acids, plays a crucial role in determining cheese yield and the structural and sensory characteristics of the final product [4]. Accordingly, whole raw Cilentana goat milk is traditionally used for the manufacture of ripened artisanal cheese, which represents one of the most characteristic dairy products associated with this indigenous breed [2]. Some studies have investigated the microbiological and physicochemical characteristics of Italian artisanal cheeses produced from raw goat milk. Dalzini et al. [5] monitored “Formaggella di capra” during manufacture and 30 days of ripening, reporting lactic acid bacteria as the predominant microbial group and variability among cheesemaking replicates. Pino et al. [6] evaluated Nicastrese raw goat milk and 60-day-ripened cheese over the production season, reporting that most microbial groups decreased over the investigated period in ripened cheese. However, information on the changes occurring during extended ripening of Italian raw goat-milk cheeses remains limited, particularly when microbiological, physicochemical, and lipid-related parameters are considered together. However, information on the changes occurring during extended ripening of Italian raw goat-milk cheeses remains limited, particularly when microbiological, physicochemical, and lipid-related parameters are considered together. Therefore, the aim of this research was to study the evolution of the physicochemical and microbiological characteristics of raw milk Cilentana goat cheeses during the cheese-making and ripening period.

## 2. Materials and Methods

### 2.1. Sampling Procedures

The experimental study was conducted in a dairy farm located in Cilento (Campania, Italy) between March and October 2023. Four independent batches (*n* = 4) of Cilentana goat cheese were produced in April, May, June, and July 2023, yielding a total of thirty-two cheeses (eight cheeses per batch). For each batch, the sampling plan included 100 mL of bulk milk, 300 g of curd and two cheeses collected on the day of cheese production (0 d). During ripening, two cheeses per batch were collected at each sampling time, namely at 30, 60, and 90 days of ripening. At each sampling time, one of the two collected cheeses was assigned to microbiological analyses, while the other was used for physicochemical analyses. After collection, all the samples were transported in refrigerated containers (4 ± 1 °C) to the Food Inspection Laboratories of the Department of Veterinary Medicine and Animal Production at the University of Naples Federico II for microbiological and physicochemical analyses.

### 2.2. Manufacturing Technology of Cilentana Goat Cheese

The traditional goat cheeses investigated in the present study were produced from fresh raw goat milk according to the manufacturing protocol provided by the local producer. Briefly, the raw milk was filtered and heated to 37 ± 1 °C, after which kid rennet (3 g L^−1^ of milk) was added. The indigenous microbiota naturally occurring in the raw goat milk contributed to milk acidification, thereby supporting the rennet-induced coagulation process. The milk was allowed to coagulate for 25 min. The resulting curd was manually cut into clots as big as rice and left to rest for about 15 min. The curd was then transferred into cylindrical molds (102 mm internal diameter × 50 mm height) and allowed to drain at room temperature for approximately 30 min. Subsequently, the cheese wheels, while still in the molds, were briefly immersed in hot whey at approximately 80 °C to promote surface sealing. After removal from the whey, the cheeses were left to rest for 5–8 h and subsequently dry-salted by hand on the surface with coarse-grained salt for 2 days. During the salting period, the cheese wheels were turned every 2–3 h to promote uniform salt distribution throughout the cheese. After salting, the cheeses were immersed again in hot whey and then allowed to dry on racks before ripening. Finally, the cheeses were transferred to a controlled ripening chamber maintained at 8 ± 1 °C and 84–90% relative humidity (RH) where they were ripened for 90 days and turned every 1–3 days to promote uniform ripening.

### 2.3. Physicochemical and Fatty Acid Analysis

The chemical composition and the fatty acid (FA) profile of cheeses were determined from grated samples taken from a representative section of each cheese obtained by collecting the matrix 2 cm from the rind. The contents of protein (AOAC Official Method 992.15), salt (AOAC Official Method 960.29), fat (AOAC Official Method 960.39) and moisture (AOAC Official Method 950.46), expressed as percentages (%), were determined following the methods outlined by the Association of Official Analytical Chemists [7].

pH and water activity (a_w_) values were measured throughout the cheese-making and ripening processes using a digital pH meter (Crison-Micro TT 2022, Crison Instruments, Barcelona, Spain) and an Aqualab 4 TE (Decagon Devices Inc., Pullman, WA, USA) instrument, respectively. The extraction of fat for the subsequent gas chromatographic (GC) analysis was carried out according to the Official Method of Cheese Analysis [8], based on the Schimith–Bondzynski–Ratzlaff traditional method of extracting lipids. Derivatization to determine the composition of fatty acids (FAs) was performed on milk, curd and cheeses lipids extract following the method described by Di Paolo et al. [9]. An aliquot (1 μL) of the extract was injected into a DANI Master GC (DANI Instruments S.p.A., Cologno Monzese, Italy), equipped with a flame ionization detector (FID), a split/splitless injector, and a CP-Select CB capillary column (Varian, Middelburg, The Netherlands) for FAME analysis (100 m × 0.25 mm i.d., 0.20 μm film thickness). Both the injector and detector temperatures were maintained at 250 °C. Helium was used as carrier gas at a flow rate of 1.2 mL/min. The oven temperature was initially set at 140 °C and held for 5 min, then increased to 240 °C at a rate of 4 °C/min and maintained at the final temperature for 18 min.

Fatty acid methyl esters were identified by comparing the retention times of the peaks in the sample with previously run pure standard compounds (Supelco^®^ 37 Component FAME Mix, 47885U-Supelco, Sigma-Aldrich, St. Louis, MO, USA). Moreover, conjugated linoleic acid (CLA) isomers were identified using an external standard (Linoleic acid, conjugated methyl ester, O5632, Sigma-Aldrich, MI, USA, IT). Fatty acids were quantified by relative peak area normalization and expressed as a percentage of total identified fatty acids. To assess the lipid quality indices in ripened cheeses [10,11], the index of atherogenicity (AI) and thrombogenicity (TI), health-promoting index (HPI) and hypocholesterolemic/hypercholesterolemic (h/H) ratio were calculated based on the FA composition, as follows:

(1) $$\mathrm{AI}=\left[C12:0+\left(4\times C14:0\right)+C16:0\right]/(n3\;\mathrm{PUFA}+n6\;\mathrm{PUFA}+\sum \mathrm{MUFA})\;$$

(2) $$\mathrm{TI}=\left(C14:0+C16:0+C18:0\right)/\left[(0.5\times \Sigma \mathrm{MUFA}+0.5\times \mathrm{PUFA}\;\left(n6\right)+\left(n3\right)/\left(n6\right)\right]$$

(3) $$\mathrm{HPI}=\mathrm{PUFA}\;\left(n6\right)+\mathrm{PUFA}\;\left(n3\right)+\Sigma \mathrm{MUFA}/\left[(12:0+\left(4\times C14:0\right)+C16:0\right]$$

(4) $$h/H=\left(C18:1n9cis+\Sigma \mathrm{PUFA}\right)/\left(C12:0+C14:0+C16:0\right)$$

### 2.4. Lipolysis, Oxidative Stability and Color Analysis of Cheeses

The lipolysis and oxidative stability during cheese-making and ripening processes were evaluated by measuring the concentration of free fatty acids (FFAs) and that of thiobarbituric acid reactive substances (TBARs) following the methods described by Di Paolo et al. [9]. The results were expressed as the percentage (%) of free oleic acid present in the sample for FFAs and as mg of malondialdehyde (MDA), which is a secondary oxidation product, per kg of sample for TBARs. According to Ambrosio et al. [12], color evaluation (CIEL*a*b*) was performed on 3 cm thick slices using a Konica Minolta CR300 colorimeter (Minolta, Osaka, Japan). For each sample, color measurements were performed on internal surfaces, with three readings recorded for each one.

### 2.5. Microbiological Analyses

Milk samples (10 mL) and curd/cheese samples (10 g) were aseptically transferred into stomacher bags containing 90 mL of sterile Peptone Water (PW) (1:10 *w*/*v* or *v*/*v*) and homogenized using a peristaltic homogenizer (BagMixer^®^400 P, Inter science, Saint-Nom-la-Bretèche, France) for 2 min at 230 rpm. Sequential decimal dilutions of the homogenates were prepared in 0.1% PW and plated in duplicate. Microbial enumeration was performed using the following selective media and incubation conditions: Total Aerobic Bacteria on Plate Count Agar incubated at 30 °C for 72 h (TAB 30 °C) (ISO 4833-1:2013) [13]; coliforms in Violet Red Bile Agar incubated at 37 °C for 24 h; β-glucuronidase-positive *E. coli* in Tryptone Bile X-Glucuronide Agar incubated at 44 °C for 24/48 h (ISO 16649-2:2010) [14]; coagulase-positive staphylococci on Baird Parker Agar supplemented with Egg Yolk Tellurite Emulsion incubated at 37 °C for 24/48 h (ISO 6888-1:2021) [15]; mesophilic lactococci on Medium 17 Agar supplemented with 5 g/L of lactose incubated aerobically at 30 °C for 48 h [16]; mesophilic lactobacilli on De man, Rogosa and Sharpe (MRS) agar incubated anaerobically at 37 °C for 72 h [17]; enterococci on Slanetz–Bartley agar incubated at 45 °C for 48 h [18].

The surface-spread technique (0.1 mL) was used for all the media, except for TBX and VRBA, where the pour plate technique (1 mL) or the pour plate with overlay technique was utilized, respectively. All the media were purchased from Oxoid (Basingstoke, UK). The results were expressed as mean values and standard deviations and reported in Log CFU/g.

In addition to microbial enumeration, each sample was examined for the presence of *Salmonella* spp. and *Listeria monocytogenes* in 25 g/mL using culture enrichment, following the respective reference methods (ISO 6579-1:2017 and ISO 11290-1:2017) [19,20].

All the presumptive colonies of *L. monocytogenes*, *Salmonella* spp., and coagulase-positive *Staphylococcus aureus* were confirmed by MALDI-TOF mass spectrometry (MALDI Biotyper^®^ Sirius, Bruker Corporation, Bremen, Germany) using the direct sample spotting technique as reported previously by Mazzocca et al. [21].

### 2.6. Statistical Analysis

Statistical analyses were performed using SPSS software, version 29 (IBM Analytics, Armonk, NY, USA). Microbiological data as well as physicochemical and color parameters (*n* = 4) were statistically analyzed using a generalized linear mixed model (GLMM), including ripening time as a fixed effect and production batch as a random effect. Means were compared using Tukey’s test (*p* < 0.05; *p* < 0.01). All data are presented as mean (M) ± standard deviation (sd).

## 3. Results and Discussion

### 3.1. Microbiological Profile

The main microbial groups were monitored throughout cheesemaking and ripening (Table 1). Considering that the safety and microbiological quality of raw milk can significantly influence the composition and characteristics of the final product [22], the microbial profile of raw milk was also assessed. Neither *Salmonella* spp. nor *Listeria monocytogenes* were detected in any of the samples analyzed [23].

The mean TAB 30 °C count in raw goat milk was 6.15 ± 0.74 Log CFU/g. This value is slightly lower than those reported in other studies on raw goat milk (~7 Log CFU/g) by Alonso Calleja et al. [24] and Pino et al. [6], yet marginally higher than the counts (~5 Log CFU/g) reported by Dalzini et al. [5] and Gonzalez et al. [25]. The TAB 30 °C level measured in the present study complies with the regulatory limits established by Regulation (EC) No. 853/2004 [26], which restricts raw milk from goats and ewes to a maximum of 1,500,000 CFU/g if it is intended for heat-treated dairy products, and 500,000 CFU/g for raw-milk cheeses ripened no longer than 60 days [27]. TAB 30 °C enumeration is a widely used indicator of the hygienic quality of raw milk, as it reflects the overall level of microbial contamination associated with milking practices, equipment hygiene, and storage conditions. During cheesemaking, the TAB 30 °C count exhibited only a marginal increase to 6.60 ± 0.24 Log CFU/g in cheese at 0 day. However, a significant increase (*p* < 0.05) of approximately 2 log units was observed within the first month of ripening, reaching approximately 8.23 ± 0.15 Log CFU/g.

This trend is consistent with previous findings on traditional goat cheeses, such as Formaggella di capra [5], Majorero [28], and Tenerife cheese [29], all demonstrating similar increases after 30 days of ripening. Thereafter, TAB counts remained relatively stable throughout the remainder of the ripening period (up to 90 days). High levels of coliforms (4.73 ± 1.80 Log CFU/mL) were detected in raw goat milk. These bacteria are widely acknowledged as indicators of the hygienic-sanitary conditions of dairy production, and their enumeration provides valuable insight into contamination events occurring during or after milking and early processing stages [30,31]. The mean values remained relatively constant, ranging from 4.27 ± 0.96 to 5.47 ± 0.07 Log CFU/g, between the curd and cheeses sampled at day 0, as well as throughout the entire ripening period. These counts are consistent with those previously reported for traditional goat cheeses, including Caprino d’Aspromonte [18], Majorero [25], and the cheese studied by Fontecha et al. [28]. However, in contrast to the latter, who reported significant reductions in coliforms at 60 and 90 days of ripening, no such decrease was observed in the present study. From a technological and public health standpoint, high coliform levels represent a critical issue for the dairy industry. Although coliforms are not considered inherently pathogenic, their presence raises concerns as they may signal the potential presence of more dangerous microorganisms. The persistence of coliforms observed in the current study may be partially attributed to the technological and physicochemical properties of the cheese. Notably, the pH remained relatively high (5.32–5.37) throughout ripening, with values significantly above the level (<5.0) typically associated with coliform inhibition [31]. Additionally, the salt content reached inhibitory levels only after 30 days of ripening, increasing from 0.62% at day 0 to 4.41%, suggesting that coliforms had sufficient time to establish before effective osmotic stress. The water activity also remained favorable for microbial growth (≥0.95) up to day 60, decreasing to 0.86 only at the final stage of ripening, consistent with its recognized role in coliform survival in ripened cheeses [32]. Moreover, the use of thermized milk without the addition of starter cultures may have limited the early growth of competitive lactic acid bacteria (LAB), thereby reducing microbial antagonism and ecological pressure on coliform populations [33,34]. These combined factors likely contributed to a permissive environment enabling coliform persistence during ripening. These results highlight the critical role of initial milk hygiene and early processing parameters in determining the microbial profile of raw- or thermized-milk cheeses. Furthermore, they emphasize the importance of optimizing ripening conditions, particularly pH decrease, salt diffusion, and microbial competition, to ensure the hygienic safety of traditional goat-milk cheeses. Importantly, the coliform counts observed here should not be interpreted as a direct measure of consumer health risk, because generic coliform levels in cheese do not reliably predict the presence of foodborne pathogens [35,36]. Nevertheless, their persistence at approximately 4–5 Log CFU/g in a raw-milk cheese indicates suboptimal hygienic quality and warrants attention, particularly because raw milk may introduce Enterobacteriaceae from fecal, environmental, or milking-related sources [35,36]. In the present study, *Salmonella* spp. were specifically investigated, and β-glucuronidase-positive *E. coli* were enumerated; however, no targeted characterization of pathogenic *E. coli* or other pathogenic Enterobacteriaceae (e.g., STEC) was performed. Therefore, the absence of these hazards cannot be inferred from the coliform results alone, and pathogen-specific testing should be considered in future studies. Preventive measures should primarily focus on improving udder and teat hygiene, cleaning and sanitation of milking and cheesemaking equipment, rapid cooling and appropriate storage of raw milk, and systematic control of food-contact surfaces and other environmental contamination points throughout processing.

Like coliforms, *E. coli* count is usually used as an indicator of raw milk quality and hygienic conditions maintained during cheese production [37,38]. The presence of *E. coli* is considered undesirable due to potential pathogenicity of some strains and their contribution to quality defects such as texture irregularities, gas production, and off-flavors [39].

In the present study, *E. coli* (β-glucuronidase-positive) counts in raw goat milk were 3.09 ± 1.32 log CFU/g and increased slightly during cheesemaking to 3.59 ± 0.36 Log CFU/g in cheese at day 0. Although this value exceeds the threshold of 10^3^ CFU/g established by Regulation (EC) No. 2073/2005 [23] for cheeses produced from heat-treated milk, it remains close to the limit. These results suggest acceptable hygiene conditions without evidence of severe contamination. During ripening, the mean count progressively decreased, dropping below the detection limit (<10 CFU/g) starting by day 60. This trend is consistent with previous studies reporting similar reductions in *E. coli* during ripening of semi-hard raw goat-milk cheeses, which have been attributed to acidification and microbial competition. In the present study the inhibition of *E. coli* may be associated with the growth of competitive microorganisms such as lactic acid bacteria [40], as well as with significant increases in NaCl concentration [29]. Ripening is widely recognized as a natural selective process, during which lactic acid bacteria exert inhibitory effects on pathogenic microorganisms. Strains of *Lactobacillus paracasei* subsp. *paracasei* isolated from Caprino d’Aspromonte cheese have been shown to display strong antagonistic activity against *E. coli*, primarily through the production of bacteriocins [18].

The enumeration of coagulase-positive staphylococci is established as a process hygiene criterion of cheese under Regulation (EC) No. 2073/2005 [23]. In the present study, coagulase-positive staphylococci were detected in raw milk at a mean concentration of 2.55 ± 2.17 Log CFU/g, exhibiting substantial variability among samples reflected by the respective standard deviation. The highest mean value was recorded in the curd (3.43 ± 1.64 Log CFU/g), while counts in cheese samples remained below the regulatory threshold of 10^3^ CFU/g. A significant (*p* < 0.01) decrease in coagulase-positive staphylococci was observed during ripening, with mean levels decreasing from 2.59 ± 1.88 Log CFU/g at 30 days to below 100 CFU/g by 60 and 90 days. This trend is consistent with previous findings in Tenerife Cameros, Majorero and Gredos cheese, where a similar decline during maturation was reported [28,29].

Species-level identification of coagulase-positive staphylococci by MALDI-TOF MS revealed the presence of *S. aureus* in all the raw milk samples, except for the one collected in April. In addition, *S. chromogenes* and *S. simulans* were also identified in the milk. *S. aureus* was further detected in the curd and in the 0-day cheese obtained from contaminated milk batches, as well as in the 30-day ripened cheese produced in May. These findings indicate the persistence of the contaminant during the early stages of cheesemaking and ripening. Although average levels of coagulase-positive staphylococci remained below the threshold associated with staphylococcal enterotoxin production, the detection of *S. aureus* in cheeses at 0 and 30 days of ripening is noteworthy. According to the European Food Safety Authority and the European Centre for Disease Prevention and Control [41], *S. aureus* remains a relevant hazard in dairy products due to its involvement in foodborne poisoning. Therefore, its presence calls for the implementation of improved handling and sanitation practices throughout the dairy production chain.

Lactic acid bacteria (LAB) were dominant and at high levels throughout the ripening process. Autochthonous LAB, owing to their versatile enzymatic systems that enable efficient utilization of milk nutrients, are well adapted to grow under the harsh environmental conditions typically encountered during cheese ripening, such as low pH, high salt concentration, reduced water activity, and declining moisture content [42]. LAB counts were determined in different ways. Both mesophilic lactobacilli and enterococci showed an increasing trend from milk to curd.

Specifically, mesophilic lactobacilli increased from 3.31 ± 3.23 Log CFU/g in milk to 5.05 ± 0.76 Log CFU/g in cheese at 0 days, reaching 7.52 ± 1.09 Log CFU/g by day 90 of ripening. This trend contrasts with that observed in Majorero cheese, in which a 2-log reduction in this group was reported after 90 days of maturation [28]. Lactobacilli exhibit a slower metabolic rate compared to lactococci and therefore their initial growth during ripening is more limited. However, lactobacilli are commonly associated with the secondary microbiota of cheese and may play an important role during ripening [43]. Their metabolic activity can contribute to the biochemical and microbiological changes occurring throughout the ripening process, thereby influencing the overall characteristics and quality of the final product [44].

Enterococci reached their maximum concentration in the curd and remained stable (~5 Log CFU/g) throughout the ripening period, in agreement with findings from previous studies [29,45,46]. Their persistence during cheesemaking and ripening is largely attributed to intrinsic resistance to elevated temperatures, as well as to high concentrations of salt and acid [47]. Although enterococci are part of the natural microbiota of cheeses made from raw or thermized milk, their technological relevance and safety implications remain debated [48]. On the one hand, enterococci are often considered indicators of suboptimal hygienic conditions during milk processing and cheese production; on the other, they may play a beneficial role in traditional cheeses, contributing to ripening and the development of characteristic flavor profiles [49].

Mesophilic lactococci represented the most abundant microbial group, with counts approximately 3 Log units higher than other microbial groups in raw goat milk and about 1 log unit higher in the curd, with mean values around 6 Log CFU/g. Their concentrations in milk were comparable to those reported in Armada, Tenerife, Cendrat del Montsec, and Majorero cheeses [28,29,45]. However, lower levels were observed in the curd compared to those of the aforementioned studies. Lactococci peaked at 8.25 ± 0.73 Log CFU/g at 60 days of ripening, confirming their pivotal role in acidification and in maintaining microbial balance during maturation. Their predominance in the early stages of ripening is attributed to their rapid metabolic activity, which enables efficient lactose fermentation and prompt proliferation shortly after curd formation [24].

Overall, the microbiological profile of the cheese reflected dynamic shifts during ripening, characterized by a decline in hygiene indicators and spoilage microorganisms such as *E. coli* and coagulase-positive staphylococci and the progressive dominance of LAB groups essential for fermentation and the development of sensory attributes. The findings highlight the need to improve the hygienic quality of goat cheese. It can be stated that from milk to curd there is no substantial increase in microbial groups, with the exception of LAB. This may suggest that no contamination occurred during curd handling or cheese production, and that the issue is primarily related to the initial microbial load of the milk. In this context, enhancing milking hygiene practices and milk storage conditions could contribute to a higher microbiological standard for this distinctive artisanal product. This study provides detailed insights into the microbiological dynamics associated with the ripening of goat cheese. To the best of our knowledge, there is no extensive literature documenting the microbial evolution of thermized Cilentana goat-milk cheeses over a 90-day ripening period.

### 3.2. Physicochemical Parameters of Cilentana Goat Cheese

From a chemical perspective, the bulk raw milk from Cilentana goats used for cheesemaking contained 4.30% fat and 3.78% protein. The physicochemical and compositional changes observed during cheesemaking and ripening are summarized in Table 2. Overall, the results provide a detailed chemical and nutritional characterization of Cilentana goat cheese and show progressive changes in its composition during ripening. These changes may be associated with the primary and secondary biochemical processes typically occurring during cheese ripening, including proteolysis, lipolysis, residual lactose metabolism, and the subsequent metabolism of amino acids and fatty acids, as reported in previous studies [50,51].

A progressive decrease in moisture content was observed during the ripening period, mainly attributable to curd syneresis, cheese drying, and lactic microbiota activity [9,21,52]. Approximately 2% of the initial curd water content was lost during the draining process, resulting in a moisture content of about 59% in the cheese on the first day of production [9,21]. During the first 30 days of ripening, the moisture content decreased by a further approximately 10%, likely reflecting water loss under the applied ripening conditions [9]. A further significant reduction (*p* < 0.01) occurred during the subsequent stages, resulting in an overall decrease of approximately 35% by the end of ripening. This trend could be explained by the acid development and microbial (especially the lactic acid bacteria) proliferation, which enhances proteolytic enzyme activity and promotes casein solubilization and hydrolysis with consequent cheese syneresis [53]. Similar moisture evolution has been described by [54] during a 75-day ripening period and by [51] during 90 days of goat cheese maturation, confirming that extended ripening generally leads to a progressive decline in water content due to structural and biochemical modifications within the cheese matrix.

An opposite trend was registered for the fat, protein and salt contents which gradually increased over time, becoming more concentrated toward the end of the ripening process as a consequence of the moisture reduction and showing statistically significant (*p* < 0.01) differences between the initial and final stages.

The pH value exhibited a significant (*p* < 0.01) decrease during the early stages of ripening, reaching the lowest value at 30 days (5.32 ± 0.12). No substantial variation up to day 60 was observed, followed by a slight increase until the end of maturation (90 days). As reported by other authors [21,45,51], the initial pH reduction could be attributed to the metabolic activity of autochthonous lactic acid bacteria, which produce organic acids during lactose fermentation and contribute to the inhibition of undesirable secondary microflora [55]. Similar pH levels have been reported by Fresno and Alvarez [45] in 90-day ripened goat cheeses.

Water activity (*aw*) showed an overall decreasing trend during ripening, broadly paralleling the reduction in the moisture content. This behavior is consistent with the influence of compositional changes and water loss occurring during cheese maturation [9,21]. However, aw values remained stable at 0.95 (0.95 ± 0.013 and 0.95 ± 0.008 respectively) between days 30 and 60 of ripening and subsequently decreased until the end of the ripening period. Similar results have been reported in mixed cheeses ripened for 90 days [56] and in experimental goat cheeses after 75 days of ripening [54].

### 3.3. Fatty Acid Profile of Milk and CILENTANA GOAT CHEESE

The fatty acid (FA) composition of Cilentana goat milk, curd and cheeses during the cheese-making and ripening period is presented in Table 3. Palmitic acid (C16:0), oleic acid (C18:1 n-9 cis), myristic acid (C14:0), capric acid (C10:0), and stearic acid (C18:0) were the predominant fatty acids, in agreement with the profile reported by Sadvaeri et al. [57] for hard cheeses made from raw goat milk. According to Mazzocca et al. [21] cheese-making did not significantly affect the FA composition of cheeses compared to the milk, confirming that coagulation and curd formation have a negligible effect on the lipid fraction. Among the FA, capric acid (C10:0), an important contributor to the typical sensory properties of goat cheeses [58,59], showed a slight decrease (*p* > 0.05) during cheese manufacture and subsequently remained relatively constant during ripening. These modest changes may reflect lipolytic activity, which promotes the release of free fatty acids from milk triglycerides. These free fatty acids are subsequently metabolized into volatile compounds that contribute to the development of the typical flavor of ripened goat cheeses [60,61].

**Figure 1:**
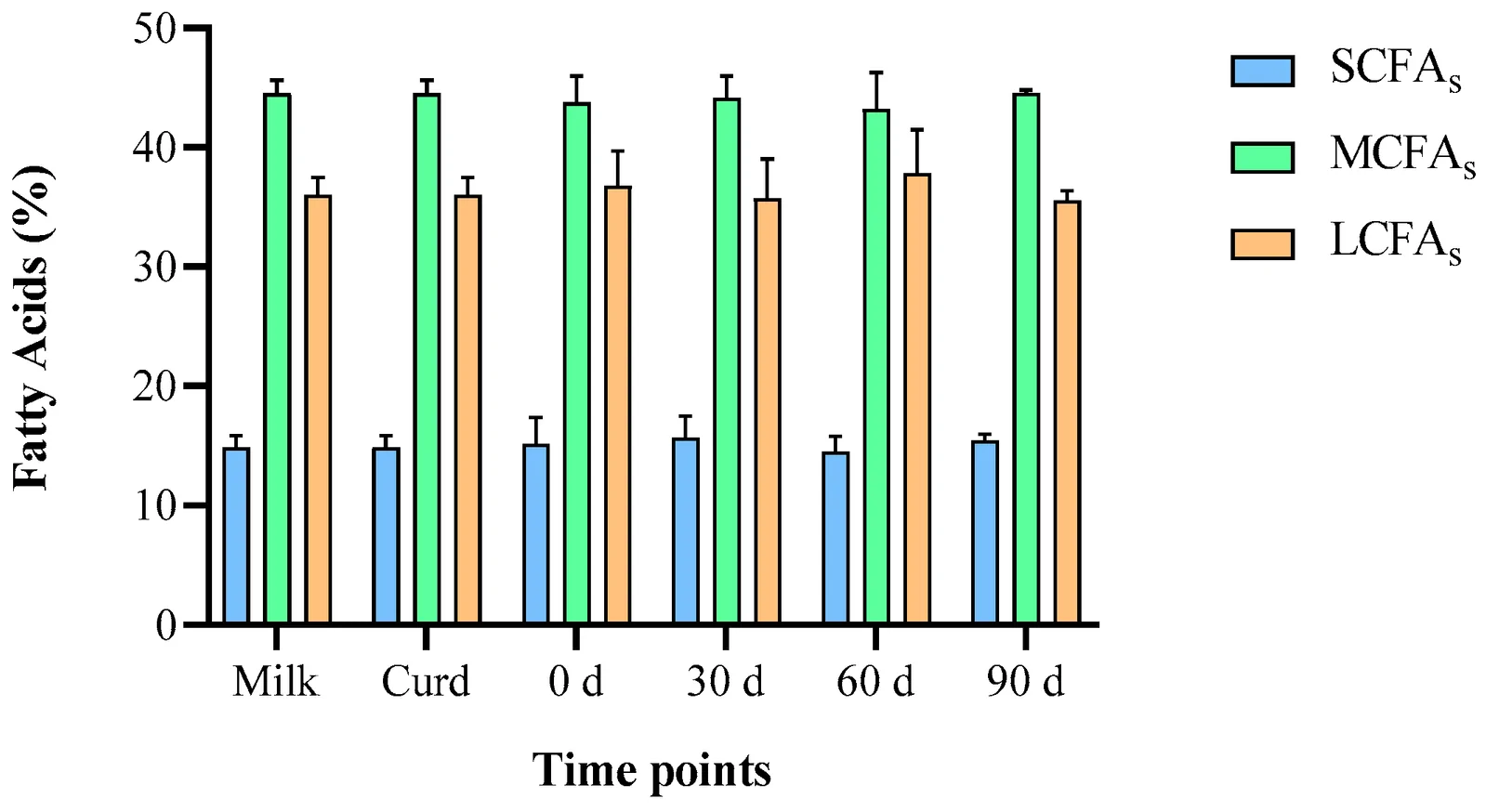
Changes in content of short-chain fatty acids, SCFAs (blue bars); medium-chain fatty acids, MCFAs (green bars); and long-chain fatty acids, LCFAs (orange bars) at each time point during cheese-making and ripening of Cilentana. SCFAs, short-chain fatty acids (C4–C10); MCFAs, medium-chain fatty acids (C12–C16); LCFAs, long-chain fatty acids (C18:0–C20:0). No statistically significant differences were observed between the FA groups at any time point (*p* > 0.05) (*n* = 4).

Only slight variations were observed in the FA profile during the ripening period, mainly involving short-chain fatty acids (SCFAs) during the first 30 days of ripening [21,57], as shown in Figure 1. Butyric acid (C4:0) decreased from 1.45% in milk to 1.22% after 30 days of ripening, while caproic (C6:0) and caprylic (C8:0) acids showed similar, although less pronounced, reductions in agreement with the observations of Correddu et al., [62]. However, these changes were limited, and no consistent temporal trend was observed during the subsequent stages of ripening. Overall, ripening up to 90 days did not substantially modify the major fatty acids. Palmitic acid (C16:0), the predominant fatty acid, showed only a slight (*p* > 0.05) decrease from 29.30% on the first day of production to 28.45% after 90 days of ripening. Oleic acid (C18:1 n-9 cis) also remained remarkably stable, whereas stearic acid (C18:0) exhibited modest fluctuations, with slightly higher values at 60 days followed by a decrease at the end of ripening, although these differences were not statistically significant. These findings differ from those reported by Di Paolo et al. [9], who observed more pronounced modifications in C18 fatty acids during prolonged ripening, suggesting that the shorter maturation period adopted in the present study had only a limited impact on the lipid composition.

The stability of individual fatty acids was further supported by the distribution of the main fatty acid classes. Saturated fatty acids (SFAs) represented the predominant fraction, accounting for approximately 74–75% of total fatty acids, followed by monounsaturated fatty acids (MUFAs; 21–22%) and polyunsaturated fatty acids (PUFAs; 3.6–4.0%). No significant differences (*p* > 0.05) were detected among milk, curd and ripened cheeses, indicating that neither cheese-making nor ripening substantially altered the overall fatty acid profile. Similar stability has been described for other traditional goat cheeses, where limited lipolysis during ripening did not produce substantial changes in the relative proportions of SFAs, MUFAs and PUFAs [9,21,57].

As regards the nutritional indices, neither cheese-making nor ripening significantly affected the nutritional quality of the lipid fraction (Table 4). The contents of n-3 and n-6 fatty acids remained relatively constant during cheese-making and ripening. Likewise, the atherogenicity (AI) and thrombogenicity (TI) indices showed only minor numerical fluctuations, and since both indices are directly influenced by the relative proportions of saturated and unsaturated fatty acids, their stability is consistent with the limited changes observed in the overall fatty acid profile. Similar results have been reported for other traditional goat cheeses, in which ripening had little effect on the nutritional quality of milk fat [21,57]. Overall, both indices demonstrated positive health attributes, as reported by Chen and Liu, and Beata Paszczyk et al. [10]. At the same time, both the hypocholesterolemic/hypercholesterolemic ratio (h/H) and the health-promoting index (HPI) also remained essentially unchanged during ripening. Although slightly higher values were observed after 60 days of maturation, these differences were not statistically significant. From a nutritional perspective, the stability of AI, TI, h/H and HPI indicates that the cheese-making process preserved the nutritional quality of Cilentana goat cheeses regardless of the ripening time. This finding suggests that the biochemical changes occurring during ripening were insufficient to substantially modify the nutritional quality of the fat fraction, confirming the interesting nutritional value of this traditional goat cheese.

### 3.4. Lipolysis and Oxidative Stability in Cilentana Goat Cheese

Thiobarbituric acid reactive substances (TBARs) and free fatty acids (FFAs) were monitored as indicators of lipid oxidation and lipolysis, respectively. Their evolution during cheese ripening may be influenced by several compositional factors, including the fatty acid profile and the presence of endogenous antioxidant compounds [21]. A numerical increase in TBARs values was observed (Table 5) during ripening, although differences were not statistically significant (*p* > 0.05). An increase in TBA levels can be observed at 90 days of ripening (0.174 ± 0.193). Overall, the TBARs values remained low and were comparable to those reported by Mazzocca et al. for Pecorino Bagnolese cheese. Although lipid oxidation represents a major quality concern in dairy products, particularly during ripening [63], the low TBARs values observed in this study indicate limited oxidative deterioration, likely due to the presence of natural antioxidant components in goat milk and the protective effect of the cheese matrix. In cheese, free fatty acids (FFAs) released through lipolysis, especially short- and medium-chain fatty acids, play a key role in flavor development [60]. Our results showed a gradual increase during ripening, with a significant difference observed after 90 days of ripening (*p* < 0.01).

### 3.5. Color Parameters of Cilentana Goat Cheese

The effects of ripening on curd and cheese color are reported in Table 6. Color evaluation and its evolution during goat cheese maturation are important indicators of physicochemical changes occurring within the cheese matrix and, consequently, of product quality. As previously reported by several authors [64,65,66], our results indicate a significant decrease (*p* < 0.01) in the lightness parameter (L*) from the beginning of ripening (90.79 ± 0.15) to day 90 (69.50 ± 0.73). This reduction may be related to water loss and the consequent increase in the relative concentration of proteins, lipids, and endogenous pigments, which can reduce light scattering within the cheese matrix [65]. The a* parameter did not exhibit significant variation throughout the ripening period, indicating that redness was not influenced by the biochemical or physical changes occurring during maturation. In contrast, the yellowness parameter (b*) showed a significant increase from day 30 onwards until the end of maturation. Comparable behavior was reported by Macias et al. [66] who observed similar color parameters in traditional goat cheese over 28 days of ripening. The progressive increase recorded during the later stages of maturation may reflect changes in the cheese, related to lipid oxidation and other ripening-associated biochemical reactions [67]. Chroma* also increased significantly by day 30 and remained higher throughout the subsequent stages of ripening. Since no significant changes were detected in the a* parameter, the increase in color saturation was attributable to the greater yellow component. Moreover, the negative a* values indicate that ripening did not significantly affect the position of the cheese color along the green–red axis.

## 4. Conclusions

This study provides a comprehensive characterization of the microbiological, physicochemical, and lipid changes occurring during the manufacture and 90-day ripening of traditional raw milk Cilentana goat cheeses. Ripening promoted the establishment of a stable lactic acid bacteria community, accompanied by the progressive reduction in hygiene indicator microorganisms such as *Escherichia coli* and coagulase-positive staphylococci, while confirming the absence of *Salmonella* spp. and *Listeria monocytogenes*. Nevertheless, the persistence of relatively high coliform counts throughout maturation highlights the importance of improving raw milk hygienic quality and milking management practices to further enhance product safety. From a physicochemical perspective, despite the occurrence of lipolysis, the fatty acid composition and the nutritional lipid quality indices remained remarkably stable throughout ripening, indicating that the traditional manufacturing process preserves the nutritional value of the lipid fraction while ensuring limited oxidative deterioration. Overall, these findings provide new scientific evidence on the ripening dynamics of an under-investigated artisanal goat cheese, contributing to the valorization of traditional dairy products. They also underline the potential of Cilentana goat cheese as a product with distinctive microbiological and compositional traits linked to its raw milk origin and traditional production environment. The results may support the optimization of production practices aimed at improving microbiological quality while preserving the distinctive compositional and nutritional characteristics of this traditional cheese. Further studies combining sensory evaluation with metagenomic and metabolomic approaches would provide deeper insights into the relationship between microbial succession, biochemical transformations, and the development of the characteristic quality traits of Cilentana goat cheese. Further studies combining sensory evaluation with metagenomic and metabolomic approaches, and including cheeses collected from different producers, would provide deeper insights into the relationship between microbial succession, biochemical transformations, and the development of the characteristic quality traits of Cilentana goat cheese, as well as allow the variations in cheese properties to be analyzed.

## Figures and Tables

**Table 1:** Counts of microbial groups (Log CFU g^−1^) in goat milk and cheese during production and ripening (*n* = 4).

Microbial Groups	Milk	Curd	Ripening Time (Days)			
			0	30	60	90
TAB 30 °C	6.15 ± 0.74	6.56 ± 0.65	6.60 ± 0.24 ^a^	8.23 ± 0.15 ^b^	8.52 ± 0.11 ^b^	8.58 ± 0.16 ^b^
Coliforms	4.73 ± 1.80	4.27 ± 0.96	4.49 ± 0.85	5.27 ± 1.02	4.95 ± 0.59	5.47 ± 0.07
β-glucuronidase-positive *E. coli*	3.09 ± 1.32	3.26 ± 0.88	3.59 ± 0.36	2.84 ± 0.48	<10 cfu g^−1^	<10 cfu g^−1^
*Staphylococcus* spp.	5.09 ± 1.18	4.64 ± 0.29	4.73 ± 0.24	4.43 ± 0.86	5.04 ± 0.45	4.36 ± 0.57
Coagulase-positive staphylococci	2.55 ± 2.17	3.43 ± 1.64	2.65 ± 1.90 ^A^	2.59 ± 1.88 ^A^	<100 cfu g^−1 B^	<100 cfu g^−1 B^
Mesophilic lactobacilli	3.31 ± 3.23	5.36 ± 0.57	5.05 ± 0.76 ^a^	6.79 ± 0.71	5.88 ± 0.91	7.52 ± 1.09 ^b^
Mesophilic lactococci	6.10 ± 0.83	6.12 ± 0.58	5.71 ± 0.43 ^a,A^	7.74 ± 0.65 ^b^	8.25 ± 0.73 ^B^	8.07 ± 0.49 ^b^
Enterococci	3.32 ± 2.87	5.19 ± 0.78	5.11 ± 1.12	5.15 ± 1.13	5.04 ± 0.38	5.59 ± 0.36

Abbreviation: TAB 30 °C, total aerobic bacteria at 30 °C. Values are expressed as mean (M) ± standard deviation (sd). Statistical analyses were performed by comparing the parameters of milk, curd and cheese at each ripening time (0, 30, 60 and 90 days). The a,b mean values in the same row (storage time) with different letters differ significantly at *p* < 0.05 (lowercase) or *p* < 0.01 (uppercase).

**Table 2:** Chemical composition of Cilentana goat curd and cheeses during the cheese-making and ripening time (0, 30, 60, 90 days) (*n* = 4).

Items	Curd	Ripening Time (Days)			
		0	30	60	90
Moisture, %	61.39 ± 2.58 ^A^	59.66 ± 2.44 ^A^	48.09 ± 4.46 ^B^	38.14 ± 5.46 ^B^	26.75 ± 6.22 ^C^
Fat, %	15.95 ± 3.73 ^A^	18.16 ± 1.77 ^A^	20.11 ± 1.94 ^A^	27.49 ± 1.07 ^B^	31.70 ± 2.59 ^B^
Protein, %	16.33 ± 1.69 ^A^	16.77 ± 2.03 ^A^	20.65 ± 2.73 ^A^	24.48 ± 0.03 ^B^	25.35 ± 0.46 ^B^
Salt, %	0.62 ± 0.14 ^A^	0.62 ± 0.00 ^A^	4.41 ± 1.02 ^B^	4.78 ± 0.02 ^B^	6.37 ± 0.05 ^C^
pH	6.71 ± 0.03 ^A^	6.74 ± 0.02 ^A^	5.32 ± 0.12 ^B^	5.32 ± 0.35 ^B^	5.37 ± 0.06 ^B^
a_w_	0.98 ± 0.004 ^A^	0.99 ± 0.002 ^A^	0.95 ± 0.013 ^A^	0.95 ± 0.008 ^A^	0.86 ± 0.052 ^B^

The data are presented as mean (M) ± standard deviation (sd). Statistical analyses were performed by comparing the parameters of curd and cheese at each ripening time (0, 30, 60 and 90 days). The A,B,C mean values in the same row (storage time) with different letters differ significantly at *p* < 0.01.

**Table 3:** Fatty acid profile of Cilentana milk and cheeses during cheese-making and ripening (0, 30, 60, 90 days) (*n* = 4).

Fatty Acids (% of Total Fatty Acids)	Milk	Curd	Ripening Time (d)			
			0	30	60	90
C4:0	1.45 ± 0.12	1.24 ± 0.07	1.35 ± 0.24	1.22 ± 0.10	1.35 ± 0.06	1.31 ± 0.20
C6:0	2.06 ± 0.24	1.86 ± 0.16	1.99 ± 0.30	1.93 ± 0.27	1.92 ± 0.11	1.97 ± 0.24
C8:0	2.85 ± 0.13	2.48 ± 0.16	2.53 ± 0.36	2.60 ± 0.31	2.47 ± 0.12	2.62 ± 0.09
C10:0	10.91 ± 0.34	9.32 ± 0.63	9.32 ± 1.33	9.95 ± 1.12	8.78 ± 1.05	9.59 ± 0.04
C11:0	0.25 ± 0.07	0.21 ± 0.01	0.24 ± 0.06	0.27 ± 0.06	0.20 ± 0.00	0.25 ± 0.05
C12:0	4.35 ± 0.16	4.05 ± 0.56	3.65 ± 0.58	4.30 ± 0.58	3.51 ± 0.43	4.14 ± 0.62
C13:0	0.11 ± 0.01	0.10 ± 0.00	0.10 ± 0.00	0.14 ± 0.06	0.10 ± 0.00	0.15 ± 0.05
C14:0	9.88 ± 0.95	10.00 ± 0.98	9.42 ± 0.56	9.91 ± 1.02	9.46 ± 0.94	10.45 ± 0.29
C14:1	0.11 ± 0.01	0.10 ± 0.00	0.10 ± 0.00	0.10 ± 0.00	0.10 ± 0.00	0.10 ± 0.00
C15:0	1.15 ± 0.05	1.14 ± 0.11	1.18 ± 0.15	1.05 ± 0.06	1.28 ± 0.06	1.26 ± 0.15
C16:0	29.23 ± 0.97	29.15 ± 0.46	29.30 ± 1.42	28.65 ± 1.79	28.77 ± 1.71	28.45 ± 1.10
C16:1	0.64 ± 0.19	0.62 ± 0.12	0.57 ± 0.06	0.54 ± 0.06	0.61 ± 0.10	0.61 ± 0.00
C17:0	1.03 ± 0.02	1.10 ± 0.10	1.11 ± 0.17	1.05 ± 0.06	1.18 ± 0.06	1.11 ± 0.10
C17:1	0.16 ± 0.08	0.17 ± 0.05	0.24 ± 0.06	0.17 ± 0.06	0.24 ± 0.06	0.25 ± 0.05
C18:0	11.72 ± 0.08	12.90 ± 1.83	14.01 ± 1.50	12.65 ± 1.22	14.08 ± 0.73	12.76 ± 0.60
C18:1*n*-9 *trans*	1.79 ± 0.12	2.08 ± 0.38	1.76 ± 0.24	2.03 ± 0.10	1.76 ± 0.25	1.67 ± 0.26
C18:1*n*-9 *cis*	18.93 ± 2.00	18.91 ± 0.35	18.84 ± 1.45	18.91 ± 2.01	19.88 ± 2.80	18.97 ± 0.98
C18:2*n*-6 *trans*	0.42 ± 0.20	0.45 ± 0.18	0.34 ± 0.06	0.44 ± 0.16	0.27 ± 0.06	0.40 ± 0.20
C18:2*n*-6 *cis*	1.81 ± 0.25	1.72 ± 0.10	1.82 ± 0.17	1.73 ± 0.18	1.86 ± 0.25	1.77 ± 0.04
C20:0	0.28 ± 0.04	0.27 ± 0.05	0.34 ± 0.06	0.34 ± 0.06	0.37 ± 0.06	0.30 ± 0.00
C18:3*n*-3	0.73 ± 0.07	0.76 ± 0.10	0.64 ± 0.06	0.78 ± 0.15	0.59 ± 0.10	0.71 ± 0.10
CLA *cis*-9, *trans*-11	0.63 ± 0.13	0.73 ± 0.11	0.64 ± 0.16	0.74 ± 0.12	0.68 ± 0.21	0.66 ± 0.15
∑SFA_s_	75.10 ± 2.14	74.06 ± 0.93	74.82 ± 1.58	74.25 ± 2.66	73.75 ± 3.62	74.57 ± 1.52
∑MUFA_s_	21.06 ± 1.76	22.00 ± 0.73	21.54 ± 1.38	21.79 ± 2.20	22.62 ± 3.14	21.65 ± 1.15
∑PUFA_s_	3.84 ± 0.38	3.94 ± 0.26	3.65 ± 0.27	3.96 ± 0.47	3.63 ± 0.50	3.79 ± 0.37

Abbreviations: ∑SFAs, sum of saturated fatty acids; ∑MUFAs, sum of monounsaturated fatty acids; ∑PUFAs, polyunsaturated fatty acids. Values were expressed as mean (M) ± standard deviation (sd). No statistically significant differences were observed between the FA groups at any time point (*p* > 0.05).

**Table 4:** Nutritional index of Cilentana milk and cheeses during the cheese-making and ripening (0, 30, 60, 90 days) (*n* = 4).

	Milk	Curd	Ripening Time (d)			
			0	30	60	90
n-3	1.04 ± 0.04	0.97 ± 0.11	0.81 ± 0.00	0.98 ± 0.15	0.79 ± 0.10	0.91 ± 0.10
n-6	2.10 ± 0.28	2.25 ± 0.12	2.19 ± 0.21	2.23 ± 0.35	2.16 ± 0.21	2.22 ± 0.11
AI	3.09 ± 0.25	2.82 ± 0.06	2.82 ± 0.32	2.85 ± 0.45	2.72 ± 0.57	2.93 ± 0.15
TI	3.40 ± 0.28	3.37 ± 0.23	3.60 ± 0.23	3.35 ± 0.37	3.51 ± 0.57	3.45 ± 0.32
h/H	0.49 ± 0.04	0.53 ± 0.01	0.53 ± 0.06	0.54 ± 0.08	0.57 ± 0.12	0.53 ± 0.03
HPI	0.33 ± 0.03	0.35 ± 0.01	0.36 ± 0.04	0.36 ± 0.05	0.38 ± 0.08	0.34 ± 0.02

Abbreviations: n-3, omega-3 PUFA; n-6, omega-6 PUFA; AI, atherogenicity index; TI, thrombogenicity index; h/H, hypocholesterolemic/hypercholesterolemic ratio and HPI, health-promoting index. Values were expressed as mean (M) ± standard deviation (sd). No statistically significant differences were observed between the FA groups and calculated index at any time point (*p* > 0.05).

**Table 5:** Trend of TBARs and FFA values in Cilentana goat curd and cheeses during the cheese-making and ripening time (0, 30, 60, 90 days) (*n* = 4).

	Curd	Ripening Time (d)			
		0	30	60	90
TBARs, mg MDA/Kg of cheese	0.108 ± 0.088 ^A^	0.055 ± 0.07 ^B^	0.072 ± 0.101 ^B^	0.104 ± 0.08 ^A^	0.174 ± 0.193 ^C^
FFAs, % oleic acid	0.058 ± 0.023 ^A^	0.045 ± 0.008 ^A^	0.067 ± 0.02 ^A^	0.086 ± 0.03	0.157 ± 0.06 ^B^

The data are presented as mean (M) ± standard deviation (sd). Statistical analyses were performed by comparing the parameters of curd and cheese at each ripening time (0, 30, 60 and 90 days). The A,B,C mean values in the same row (storage time) with different letters differ significantly at *p* < 0.01.

**Table 6:** Trends of color parameters L*, a*, b* and Chroma in curd and cheese during ripening (0, 30, 60, 90 days) (*n* = 4).

	Curd		Ripening Time (d)		
		0	30	60	90
L*	89.77 ± 0.69 ^A^	90.79 ± 0.15 ^A^	75.79 ± 2.17 ^B^	80.08 ± 1.33 ^C^	69.50 ± 0.73 ^D^
a*	−1.23 ± 0.02 ^A^	−1.07 ± 0.08 ^B^	−1.32 ± 0.14 ^A^	−0.90 ± 0.19 ^B^	−1.06 ± 0.43 ^B^
b*	9.99 ± 0.54 ^A^	9.10 ± 0.16 ^A^	15.99 ± 0.23 ^Ba^	14.72 ± 0.42 ^Bb^	14.85 ± 0.69 ^B^
Chroma	10.07 ± 0.53 ^A^	9.16 ± 0.16 ^A^	16.04 ± 0.23 ^Ba^	14.74 ± 0.43 ^Bb^	14.89 ± 0.66 ^B^

The data are presented as mean (M) ± standard deviation (sd). Statistical analyses were performed by comparing the parameters of curd and cheese at each ripening time (0, 30, 60 and 90 days). The A,a,B,b,C,D mean values in the same row (storage time) with different letters differ significantly at *p* < 0.05 (lowercase) or *p* < 0.01 (uppercase).

## Data Availability

The original contributions presented in this study are included in the article/Supplementary Materials. Further inquiries can be directed to the corresponding author.

## Supplementary Materials

The following supporting information can be downloaded at https://www.mdpi.com/article/10.3390/foods15183226/s1. Table S1: Chemical composition of Cilentana goat curd and cheeses during the cheese-making and ripening time (0, 30, 60, 90 days) (*n* = 4).

## Abbreviations

The following abbreviations are used in this manuscript:

FA_s_	Fatty acid profile
a_w_	Water activity
AI	Atherogenic index
TI	Thrombogenic index
h/H	Ratio of hypo- and hypercholesterolemic fatty acids
HPI	Health-promoting index
MUFAs	Monounsaturated fatty acids
PUFAs	Polyunsaturated fatty acids
SFAs	Saturated fatty acids
CLA	Conjugated linoleic acid
TBARs	Thiobarbituric acid reactive substances
FFA	Free fatty acids
TAB 30 °C	Total aerobic bacteria

## References

[1] Boyazoglu J., Hatziminaoglou I., Morand-fehr P. (2005). The Role of the Goat in Society: Past, Present and Perspectives for the Future. Small Rumin. Res..

[2] Iommelli P., Infascelli L., Tudisco R., Capitanio F. (2022). The Italian Cilentana Goat Breed: Productive Performances and Economic Perspectives of Goat Farming in Marginal Areas. Trop. Anim. Health Prod..

[3] Park Y.W., Ju M., Ramos M., Haenlein G.F.W. (2007). Physico-Chemical Characteristics of Goat and Sheep Milk. Small Rumin. Res..

[4] Clark S., Sherbon J.W. (2000). Coagulation Properties of Goat Milk. Small Rumin. Res..

[5] Dalzini E., Cosciani-cunico E., Sfameni C., Monastero P., Daminelli P., Losio M.N., Varisco G., Nazionale R., Emergenti R., Romagna E. (2014). Microbiological and Physico-Chemical Changes during Manufacture of an Italian Goat Cheese Made from Raw Milk. Ital. J. Food Saf..

[6] Pino A., Liotta L., Caggia C., Chiofalo V., De Nardo F., Zumbo A., Todaro A., Randazzo L., Agricoltura D., Ambiente A. (2021). Effect of Seasonality on Physico-Chemical and Microbiological Properties of Nicastrese Milk and Artisanal Cheese. FEMS Microbiol. Lett..

[7] Horwitz W., Latimer G., AOAC International (2005). Official Methods of Analysis.

[8] Italian Ministry of Agriculture and Forestry (1986). Official Methods for the Analysis of Cheeses. Ministerial Decree of 21 April 1986. Official Gazette of the Italian Republic.

[9] Di Paolo M., Vuoso V., Ambrosio R.L., Balestrieri A., Bifulco G., Anastasio A., Marrone R. (2023). Role of Feeding and Novel Ripening System to Enhance the Quality and Production Sustainability of Curd Buffalo Cheeses. Foods.

[10] Paszczyk B., Tońska E. (2022). Applied Sciences Fatty Acid Content, Lipid Quality Indices, and Mineral Composition of Cow Milk and Yogurts Produced with Different Starter Cultures Enriched with Bifidobacterium Bifidum. Appl. Sci..

[11] Chen J., Liu H. (2020). Nutritional Indices for Assessing Fatty Acids: A Mini-Review. Int. J. Mol. Sci..

[12] Ambrosio R.L., Smaldone G., Di Paolo M., Vollano L., Ceruso M., Anastasio A., Marrone R. (2021). Effects of Different Levels of Inclusion of Apulo-calabrese Pig Meat on Microbiological, Physicochemical and Rheological Parameters of Salami during Ripening. Animals.

[13] (2013). Microbiology of the Food Chain—Horizontal Method for the Enumeration of Microorganisms. Part 1: Colony Count at 30 °C by the Pour Plate Technique.

[14] (2001). Microbiology of Food and Animal Feeding Stuffs—Horizontal Method for the Enumeration of Beta-Glucuronidase-Positive *Escherichia coli*. Part 2: Colony-Count Technique at 44 °C Using 5-Bromo-4-Chloro-3-Indolyl Beta-D-Glucuronide.

[15] (1999). Microbiology of Food and Animal Feeding Stuffs—Horizontal Method for the Enumeration of Coagulase-Positive Staphylococci (*Staphylococcus aureus* and Other Species). Part 1: Technique Using Baird-Parker Agar Medium.

[16] Mormile A., Barile M., Mercogliano R., Johansson P. (2016). Dynamics of Lactic Acid Bacteria in “Pecorino Di Tramonti”—A Ewe’s Milk Cheese—With Particular Emphasis on Enterococci: A Preliminary Study. Ann. Microbiol..

[17] Blaiotta G., Aponte M.P.P. (2017). Growth Needs and Culture Media for LAB and Dairy-Associated Species. Microbiology in Dairy Processing: Challenges and Opportunities.

[18] Caridi A. (2002). Selection of Escherichia Coli-Inhibiting Strains of Lactobacillus Paracasei Subsp. Paracasei. J. Ind. Microbiol. Biotechnol..

[19] (2017). Microbiology of the Food Chain—Horizontal Method for the Detection, Enumeration and Serotyping of Salmonella. Part 1: Detection of *Salmonella* spp..

[20] (2017). Microbiology of the Food Chain—Horizontal Method for the Detection and Enumeration of *Listeria monocytogenes* and of *Listeria* spp. Part 1: Detection Method.

[21] Mazzocca R., Di Paolo M., Peruzy M.F., Rippa A., Michele A., Santoro L., Peretti V., Marrone R., Murru N. (2024). Physicochemical and Microbiological Characterisation of a Typical Italian Raw Ewe’ s Milk Cheese: Pecorino Bagnolese. Int. Dairy J..

[22] Fusco V., Cho G., Chieffi D., Kabisch J., Fanelli F., Logrieco A.F., Böhnlein C., Franz C.M.A.P. (2020). Microbial Quality and Safety of Milk and Milk Products in the 21st Century. Compr. Rev. Food Sci. Food Saf..

[23] European Commission (2005). Commission Regulation (EC) No 2073/2005 of 15 November 2005 on microbiological criteria for foodstuffs. Off. J. Eur. Union.

[24] Alonso-calleja C., Carballo J., Capita R., Bernardo A., Garcı L., Alonso-calleja C., Capita R., Bernardo A., Garcı L. (2002). Changes in the Microflora of Valdeteja Raw Goat’s Milk Cheese throughout Manufacturing and Ripening. LWT-Food Sci. Technol..

[25] Daniel I., González R., Elisa S., Elvira M., Andrade L.H. (2025). Characterization of Family Goat Farms and Determination of Risk Factors Associated with the Sanitary Qualities of Raw Milk and Fresh Cheese in Three Production Areas in Mexico. Vet. World.

[26] Council of the European Union (2004). Regulation (EC) No 853/2004 of the European Parliament and of the Council of 29 April 2004 laying down specific hygiene rules for food of animal origin. Off. J. Eur. Union.

[27] State-Regions Conference (2006). Agreement of 16 November 2006 between the Italian Government, the Regions and the Autonomous Provinces of Trento and Bolzano on adaptations for the production of cheeses with a maturation period of at least 60 days, produced from sheep’s and goat’s milk, and derogations for milk produced during the summer mountain grazing period (Repertory No. 2673). Off. Gaz. Ital. Repub..

[28] Fontecha J., Pelaez C., Juarez M., Requena T., Universitaria C., Ramos M. (1990). Artisanal Hard Goat’ s Cheese. J. Dairy Sci..

[29] Zárate V., Belda F., Pérez C., Cardell E. (1997). Changes in the Microbial Flora of Tenerife Goats’ Milk Cheese During Ripening. Int. Dairy J..

[30] Montel M., Buchin S., Mallet A., Delbes-paus C., Vuitton D.A., Desmasures N., Berthier F. (2014). International Journal of Food Microbiology Traditional Cheeses: Rich and Diverse Microbiota with Associated Bene Fi Ts. Int. J. Food Microbiol..

[31] Quigley L., Sullivan O.O., Stanton C., Beresford T.P., Ross R.P., Fitzgerald G.F., Cotter P.D. (2013). The Complex Microbiota of Raw Milk. FEMS Microbiol. Rev..

[32] Callon C., Arliguie C., Montel M.-C. (2016). Control of Shigatoxin-Producing Escherichia Coli in Cheese by Dairy Bacterial Strains. Food Microbiol..

[33] Amico D.J.D. (2014). Microbiological Quality and Safety Issues in Cheesemaking. Cheese Microbes.

[34] Camargo A.C., Paulo J., De Araújo A., Carvalho A.F.D., Nero L.A. (2021). Microbiological Quality and Safety of Brazilian Artisanal Cheeses. Braz. J. Microbiol..

[35] Trmčić A., Chauhan K., Kent D.J., Ralyea R.D., Martin N.H., Boor K.J., Wiedmann M. (2016). Coliform detection in cheese is associated with specific cheese characteristics, but no association was found with pathogen detection. J Dairy Sci..

[36] Caridi A., Micari P., Foti F., Ramondino D., Sarullo V. (2003). Ripening and seasonal changes in microbiological and chemical parameters of the artisanal cheese Caprino d’Aspromonte produced from raw or thermized goat’s milk. Food Microbiol..

[37] Giammanco G.M., Pepe A., Aleo A., Agostino V.D., Milone S., Mammina C. (2011). Microbiological Quality of Pecorino Siciliano “Primosale” Cheese on Retail Sale in the Street Markets of Palermo, Italy. New Microbiol..

[38] Metz M., Sheehan J., Feng P.C.H. (2020). Use of Indicator Bacteria for Monitoring Sanitary Quality of Raw Milk Cheeses—A Literature Review. J. Food Microbiol..

[39] Schirone M., Tofalo R., Visciano P., Corsetti A., Suzzi G. (2012). Biogenic Amines in Italian Pecorino Cheese. Front. Microbiol..

[40] Núñez M., Gaya P., Medina M. (1985). Influence of Manufacturing and Ripening Conditions on the Survival of *Listeria monocytogenes* in Hard Cheeses. J. Dairy Res..

[41] Food E., Authority S. (2022). The European Union One Health 2021 Zoonoses Report. EFSA J..

[42] Wouters J.T.M., Ayad E.H.E., Hugenholtz J., Smit G. (2002). Microbes from Raw Milk for Fermented Dairy Products. Int. Dairy J..

[43] Beresford T.P., Fitzsimons N.A., Brennan N.L., Cogan T.M. (2001). Recent Advances in Cheese Microbiology. Int. Dairy J..

[44] Cotter P.D., Ross R.P., Hill C. (2013). Bacteriocins—A Viable Alternative to Antibiotics ?. Nat. Rev. Microbiol..

[45] Tornadijo M.E., Fresno J.M., Bernardo A., Sarmiento R.M., Carballo J. (2020). Microbiological Changes throughout the Manufacturing and Ripening of a Spanish Goat’s Raw Milk Cheese (Armada Variety). Lait.

[46] Asa M.M., Ablab R.T., Oricheb J.M., Oaa I.R., Onzaleza J.G., Ebollob J.E.R., Áceresc P.C. (2002). Ibores Goat’ s Milk Cheese: Microbiological and Physicochemical Changes throughout Ripening. Lait.

[47] González L., Cuadrillero A.F., Castro J.M., Bernardo A., Tornadijo M.E. (2015). Selection of Lactic Acid Bacteria Isolated from San Simón Da Costa Cheese (PDO) in Order to Develop an Autochthonous Starter Culture. Adv. Microbiol..

[48] Giraffa G. (2003). Functionality of Enterococci in Dairy Products. Int. J. Food Microbiol..

[49] Franz C.M.A.P., Holzapfel W.H., Stiles M.E. (1999). Enterococci at the Crossroads of Food Safety?. Int. J. Food Microbiol..

[50] Popović-vranješ A., Pihler I., Paskaš S., Krstović S., Jurakić Ž., Strugar K. (2017). Production of Hard Goat Cheese and Goat Whey from Organic Goat’s Milk. Mljekarstvo.

[51] Moreira R.V., Costa M.P., Frasao B.S., Sobral V.S., Cabral C.C., Rodrigues B.L., Costa M.P. (2020). Effect of Ripening Time on Bacteriological and Physicochemical Goat Milk Cheese Characteristics. Food Sci. Biotechnol..

[52] Tabet E., Mangia N.P., Mouannes E., Hassoun G., Helal Z., Deiana P. (2016). Characterization of Goat Milk from Lebanese Baladi Breed and His Suitability for Setting up a Ripened Cheese Using a Selected Starter Culture. Small Rumin. Res..

[53] Nazari S.M., Mortazavi A.L.I., Hesari J. (2020). Proteolysis and Textural Properties of Low-Fat Ultra Fi Ltered Feta Cheese as in Fl Uenced by Maltodextrin. Int. J. Dairy Technol..

[54] Garcia V., Rovira S., Boutoial K., Ferrandini E., López M.B. (2016). Physicochemical, Microbiological, Textural and Sensory Changes during the Ripening of Pasteurised Goat Milk Cheese Made with Plant Coagulant (Cynara Scolymus). Int. J. Dairy Technol..

[55] Mariano J., De Oliveira C., Elisabete A., Antunes C., Felipe G., Sales C., Neves C., Costa M., Matilde A., Yago K. (2025). Influence of Autochthonous Lactic Acid Bacteria Cultures on the Microbiota and Biogenic Amine Production in Medium-Ripened Artisan Goat Cheese. Foods.

[56] Sarić Z., Žilić S., Begić M., Barać N., Barać M. (2025). Sensory Profile and Physical Properties of Kupres Cheese. Mljekarstvo.

[57] Sadvari V.Y., Shevchenko L.V., Midyk S.V., Korniyenko V.I., Slobodyanyuk N.M., Pylypchuk O.S., Naumenko T.V., Stetsiuk I.M. (2025). Regulatory Mechanisms in Biosystems Fatty Acid Profile of Artisanal Hard Cheeses Made from Raw Goat Milk during the Ripening Process. Regul. Mech. Biosyst..

[58] Chilliard Y., Ferlay A. (2004). Dietary Lipids and Forages Interactions on Cow and Goat Milk Fatty Acid Composition and Sensory Properties. Reprod. Nutr. Dev..

[59] Park Y.W. (2007). Rheological Characteristics of Goat and Sheep Milk. Small Rumin. Res..

[60] Collins Y.F., Mcsweeney P.L.H., Wilkinson M.G. (2003). Lipolysis and Free Fatty Acid Catabolism in Cheese: A Review of Current Knowledge. Int. Dairy J..

[61] Ey P.L.H.M. (2004). Biochemistry of Cheese Ripening. Int. Dairy J..

[62] Correddu F., Murgia M.A., Mangia N.P., Lunesu M.F., Cesarani A., Deiana P., Pulina G., Nudda A. (2021). Effect of Altitude of Fl Ock Location, Season of Milk Production and Ripening Time on the Fatty Acid pro Fi Le of Pecorino Sardo Cheese. Int. Dairy J..

[63] Jose D., Jose F. (2011). Proteolysis, Texture and Colour of a Raw Goat Milk Cheese throughout the Maturation. Eur. Food Res. Technol..

[64] Buffa N., Trujillo A.J., Pavia M., Guamis B. (2002). Changes in Textural, Microstructural, and Colour Characteristics during Ripening of Cheeses Made from Raw, Pasteurized or High-Pressure-Treated Goats’ Milk. Int. Dairy J..

[65] Fresno M., Álvarez S. (2012). Chemical, Textural and Sensorial Changes during the Ripening of Majorero Goat Cheese. Int. J. Dairy Technol..

[66] Sánchez-Macías D., Fresno M., Moreno-Indias I., Castro N., Morales-delaNuez A., Álvarez S., Argüello A. (2010). Physicochemical Analysis of Full-Fat, Reduced-Fat, and Low-Fat Artisan-Style Goat Cheese. J. Dairy Sci..

[67] Ferraz A.R., Pintado C.S. (2024). A Global Review of Cheese Colour: Microbial Discolouration and Innovation Opportunities. Int. J. Dairy Technol..

