# Supplementary material for: Physicochemical and Microbiological Characterization of Cilentana Goat Cheese
Source: Foods. 2026 Sep 12;15(18):3226. doi: 10.3390/foods15183226 (PMC13606744; doi:10.3390/foods15183226)
Supplement: Supplementary file 1 [file foods-15-03226-s001.zip › foods-4527655-supplementary.pdf]

Table S1: Chemical composition of Cilentana goat curd and cheeses during the cheese-making and ripening time (0, 30, 60, 90 days) (n = 4).

		Ripening time (days)			
**Items**	**Curd**	**0**	**30**	**60**	**90**
Dry matter, %	38.61 ± 2.58	40.34 ± 2.44	51.91 ± 4.46	61.86 ± 5.46	73.25 ± 6.22
Fat, % dry matter	41.53 ± 6.58	45.00 ± 2.92	39.33 ± 0.15	44.68 ± 4.47	43.28 ± 0.13
Protein, % dry matter	42.83 ± 2.56	41.47 ± 2.84	40.30 ± 1.40	38.30 ± 3.23	34.91 ± 2.07

The data are presented on a dry-matter basis.
